# Modifiable factors associated with cognitive health trajectories among Indigenous, Hispanic, Black, and White older adults: an exploratory longitudinal panel analysis of the health and retirement study with a focus on Indigenous peoples

**DOI:** 10.1016/j.lana.2025.101207

**Published:** 2025-08-12

**Authors:** Cliff Whetung, Ernest Gonzales

**Affiliations:** a University of Minnesota Medical School, Duluth, MN, USA; b New York University, Silver School of Social Work, NY, USA

**Keywords:** Cognitive health, Indigenous, Health equity, Native American, American Indian, Alaska Native, Social determinants of health

## Abstract

**Background:**

Despite elevated dementia risk, cognitive health among Indigenous older adults remains understudied. This study explored how modifiable factors were associated with cognitive health among Indigenous, Black, Hispanic, and White older adults.

**Methods:**

Using longitudinal panel data from the Health and Retirement Study (2006–2020, N = 27,327), we estimated mixed effect growth curve models to examine associations between modifiable factors and total cognitive function.

**Findings:**

This diverse sample of Indigenous (1.65%), Black (11.43%), Hispanic (9.85%), and White (77.07%) older adults had a mean age of 61 (SD = 9.66) and was 52% female. Despite being younger on average (M = 59), 3% of Indigenous respondents reported memory-related diagnoses three times the overall sample rate. Linear mixed-effect growth curve analysis revealed that Indigenous and Black older adults had similar cognitive trajectories. A college education was a protective factor for initial cognitive function (*b*_0_ = 3.09, 95% CI: 2.91, 3.27) and over time (*b* = 0.24, 95% CI: 0.15, 0.33) across ethnicity. Formal (*b* = 0.21, 95% CI: 0.15, 0.27) and informal (*b* = 0.26, 95% CI: 0.20, 0.32) volunteering were associated with slower rates of cognitive decline relative to non-volunteers, though these effects may partly reflect socioeconomic status. Among Indigenous older adults, higher education, volunteering, and fewer depressive symptoms were linked to better cognitive outcomes.

**Interpretation:**

Despite high levels of resource deprivation, Indigenous older adults demonstrate resilience that supports cognitive health. Expanding access to education and volunteering in later life through targeted social policy may enhance cognitive outcomes in Indigenous communities for generations.

**Funding:**

This research was supported by the 10.13039/100000049National Institute on Aging (R36AG078781).

**Research in context**

**Evidence before this study**

A growing body of evidence indicates that Native American and Alaska Native (Indigenous) older adults develop Alzheimer's disease and related dementias more often than non-Hispanic Whites and are at higher risk of early-onset dementia. Indigenous older adults are a marginalized, rapidly growing population that is underrepresented in cognitive health research. Many life experiences are related to later life cognitive health, including education, social engagement, substance use, chronic health conditions, and mental health. However, it is unknown how these factors are associated with the cognitive health of Indigenous older adults. Exploring the relationship between these modifiable factors and cognitive health can help to identify pathways for dementia prevention via social policy.

**Added value of this study**

To our knowledge, this study is the first to explore the longitudinal relationship between modifiable lifespan factors and cognitive health with a national sample of Indigenous older adults. Our findings indicate that Indigenous older adults endure inequities in educational attainment, income, and health but are also socially engaged and resilient.

**Implications of all the available evidence**

Our study contributes to a larger literature on lifespan dementia risk, which indicates that resolving inequities in dementia will necessitate social policy that improves educational access and bolsters opportunities for meaningful social engagement. Building a society that resolves structural inequities could eliminate disparities in later-life cognitive health for all older adults.

## Introduction

Cognitive health inequities across racial and ethnic groups are a significant public health concern. About 10% of all adults aged 65 or older in the United States are currently living with Alzheimer's disease, dementia, or mild cognitive impairment,^1^ and the total annual cost of caring for these older adults is expected to grow from about $450 million in 2020 (∼5% of all healthcare costs) to $3.3 trillion by 2060.^2^ Concurrent with rising costs associated with cognitive impairments, the population of Native American and Alaska Native older adults is expected to more over the same period.^3^ These Indigenous older adults (IOAs) are also 1.0–3.2 times more likely than non-Hispanic Whites to develop dementia.^4^, ^5^, ^6^ The primary etiologies of dementia cases among IOAs are Alzheimer's Disease and vascular brain injuries.^7^ Recent evidence also suggests that Indigenous peoples develop early-onset dementia at high rates, and thus, examining their cognitive health at younger ages is critically important.^8^ Despite this rapid growth in IOAs, they remain underrepresented in longitudinal cognitive health research.^9^ This limits our understanding of how the constellation of lifespan factors associated with dementia operate in Indigenous communities, our ability to design effective, sustainable, and culturally attuned prevention strategies, and points to an area where ethical practices in cognitive health research could be improved.^9^, ^10^, ^11^

A growing body of evidence suggests that more than 40% of dementia cases may be preventable.^12^ It also appears that older adults who decline faster in measures of global cognitive function are at elevated risk of developing these diseases.^13^ This relationship, initially posited by Stern's brain and cognitive reserve hypotheses,^14^ and later augmented by multilevel frameworks including the Lancet reports on dementia, prevention and care,^12^ suggest dementias are associated with lifespan risk and protective factors, including social determinants of health (e.g., sex,^15^ education,^16^ poverty,^17^ social engagement^18^), individual health behaviors (e.g., physical activity,^19^ smoking,^20^ alcohol consumption^21^), chronic health conditions (e.g., diabetes,^22^ hypertension,^23^ hearing loss,^24^ early-life head injury^25^), and mental health (e.g., depression^26^). However, while existing work has examined associations between modifiable lifespan factors and cognitive function among Black, Hispanic and White older adults,^27^^,^^28^ no existing research on modifiable factors associated with cognitive health has focused on Indigenous peoples within a racially and ethnically diverse cohort of older adults.

Concurrently, engagement with formal and informal volunteering activities is associated with improved cognition,^18^^,^^29^ and a slightly lower risk of dementia,^30^ even after accounting for selection bias.^31^^,^^32^ A wait-list control study on older volunteers in AARP's Experience Corps program showed gains in executive function and cortical plasticity among volunteers relative to matched controls.^33^ This study is particularly noteworthy due to its rigorous experimental design and sample, primarily comprised of African American older adults with low educational attainment, low income, and initial low scores on the Mini-Mental State Exam. It is hypothesized that volunteering necessitates cognitive, social, and physical effort,^34^ consistent with the environmental complexity and “use it or lose it” hypotheses. Similar to African Americans, IOAs report high interest in generative volunteer activities.^35^^,^^36^ This suggests that social engagement via volunteering may represent a modifiable factor for supporting the cognitive health of IOAs. However, significant gaps remain in understanding the intensity of volunteering activities among IOAs and their associations with cognitive health.^37^^,^^38^

This study integrated modifiable factors associated with later-life cognitive impairments identified by the Lancet Commission's report on dementia prevention, intervention, and care.^39^ The central aims of this study were to 1) compare adjusted rates of cognitive function over time and across Indigenous, Black, Hispanic, and White older adults, 2) characterize the prevalence of modifiable factors and associations with cognitive function among this sample, 3) bring attention to an often excluded ethnic group by examining within-group factors associated with cognitive function among Indigenous older adults, and 4) demonstrate that longitudinal analyses of modifiable factors associated with Indigenous cognitive health are viable despite “small sample sizes.”

## Methods

### Data and sample

This study employed data from 2006 through 2020 of the core and psychosocial leave-behind modules of the Health and Retirement Study (HRS). The HRS is a nationally representative longitudinal panel study of non-institutionalized older adults in the United States that collects comprehensive data on work, financial assets, psychosocial, physical health, and mental health. The initial cohort was recruited in 1992 (N = 20,000) and is replenished every six years. Participants are reinterviewed every two years. Between 2006 and 2020, 31,425 older adults participated in the HRS. The HRS has a multi-stage probability design that stratifies based on geographic region and oversamples Black and Hispanic older adults by about twice their national representation. The HRS does not oversample Native American or Alaska Native respondents.

Beginning with a total sample of 31,425 older adults, we included respondents in our analysis who had complete data on total cognitive function (1170 participants excluded), were 50+ years of age at first observation (657 participants excluded), self-identified their primary racial or ethnic identity as Native American or Alaska Native, Black, Hispanic, or White (1128 participants excluded), had cognitive health scores that qualified as normal at first observation (Telephone Interview for Cognitive Status scores of 7 or higher^40^; 1143 participants excluded), and completed a cognitive assessment at first observation without a caregiving proxy (no additional participants excluded after preceding inclusion criteria). These data were accessed initially in the spring of 2021, and this analysis was finalized in the fall of 2023 as part of Dr. Whetung's doctoral dissertation research with support from the National Institute on Aging (R36AG078781).

### Key measures

#### Dependent variable

Cognitive health was measured using the 27-point Telephone Interview for Cognitive Status (TICS-m) version administered to all HRS participants. The TICS-m measures total cognitive function using combined measures of working memory (5 points), immediate and delayed word recall (20 points), and processing speed (2 points).^41^ Lower scores indicate lower levels of total cognitive function, and scores on this measure can range from 0 to 27.^40^^,^^42^ This measure is most effective as a broad measure of cognitive function, and scores below 7 suggest the presence of dementia.^40^^,^^43^^,^^44^ The TICS-m has demonstrated improved sensitivity across race and ethnicity when models adjust for educational attainment.^43^

#### Independent variables

*Indigenous race/ethnicity*: This analysis classified any respondent who self-reported their primary race as Native American or Alaska Native alone or in combination with other racial identities as Indigenous to avoid undercounting Indigenous participants. We classified Black and White respondents similarly and coded all those who identified as Hispanic of any origin as such. Race/ethnicity variable was thus coded as Non-Hispanic White = 0, Non-Hispanic Black = 1, Hispanic = 2, Non-Hispanic Indigenous = 3. Non-Hispanic White older adults were the reference group for all regression models.

*Age* was measured in years at the date of HRS interview completion. Age was employed as our measurement of time in all models. Age was transformed to decades (age divided by 10) prior to regression analysis to improve the interpretation of intercepts and coefficients.

Guided by existing theory,^18^^,^^39^ this analysis included measures of social determinants of health (sex, education, low income), health behaviors (physical activity, smoking, alcohol consumption), social engagement (living alone, volunteering), physical health (diabetes, hypertension, hearing loss), mental health (depression, psychiatric conditions, and cognitive health (early-life head injury, dementia diagnoses).

*Social determinants of health* were measured using sex (male = 0, female = 1), educational attainment (less than high school = 0, high school degree = 1, some college = 2, college degree or more = 3), and annual income in the lowest quartile among all participants (second, third, or fourth quartile = 0, first quartile = 1).

*Health behaviors* were captured using indicators for whether respondents smoked cigarettes or consumed 21 or more alcoholic drinks per week on average. We operationalized *physical* activity using four items that asked how often respondents engaged in sports or activities with mild, moderate, or vigorous intensities. Respondents were coded inactive (0) if they never or hardly ever engaged in mild activities. Respondents who engaged in mildly energetic activities (e.g., vacuuming, laundry, home repairs), moderately energetic (e.g., gardening, cleaning the car, walking at a moderate pace, dancing, floor or stretching exercises), or vigorously energetic (e.g., running or jogging, swimming, cycling, aerobics or gym workout, tennis, or digging with a spade or shovel) were classified based on the highest level of physical activity they engaged in once per week or more (inactive = 0, mild activity = 1, moderate activity = 2, vigorous activity = 3).

*Social engagement* was measured by whether respondents were living alone (lived another person = 0 or lived alone = 1) and their level of engagement with different types of volunteering activities. Formal volunteering captured the number of hours respondents volunteered for religious, educational, health-related, or other charitable organizations in the previous year. Informal volunteering was measured by the number of hours spent helping friends, neighbors, or relatives who did not live with respondents and did not pay for the help in the previous year. For each type of volunteering, respondents who did not volunteer were coded as 0, those who volunteered for less than 100 h as 1, and those who volunteered for 100 h or more as 2.

*Physical and mental health* measures included indicators (0 = no diagnoses, 1 = current diagnosis) for hearing loss without hearing aids, current diagnoses by physicians of high blood pressure, diabetes, and any heart-related condition. Mental health was measured using an abbreviated version of the Center for Epidemiologic Studies Depression (CESD) scale containing eight items^45^^,^^46^ and an indicator for any diagnosis of “emotional, nervous, or psychiatric” issues. CESD depression scores range from 0 to 8, with higher scores indicating more severe depressive symptomatology (Chronbach's α = 0.81).

We captured a retrospective measure of early-life head injury using an indicator (0 = no, 1 = yes) for participant responses to the question, “Before you were 16 years old, did you have a blow to the head, a head injury or head trauma that was severe enough to require medical attention, to cause loss of consciousness or memory loss for some time.” Finally, we included an indicator for diagnoses of Alzheimer's disease, dementia, or any memory-related condition as a control for the onset of diagnosed cognitive pathologies (0 = no diagnosis of any memory-related condition, 1 = diagnosis of any memory-related condition).

### Analytic strategy

We first calculated weighted univariate and bivariate statistics to examine cross-sectional trends between Indigenous, Black, Hispanic, and White older adults at first observation. This study's descriptive analyses accounted for the HRS's complex multi-stage cluster design using person-level weights. Pearson correlations were calculated to test bivariate associations between all covariates included in adjusted models, and tolerance factors were inspected to assess multicollinearity between predictors. Stata v18.0 was used for all analyses.

We then tested linear, quadratic, and fractional polynomial level operationalizations of time (age) to identify these data's best-fitting trajectory structure. A random subset of 273 individuals was selected (∼1% of the total sample), and scatter plots of their total cognition and age were visually inspected before fitting individual-level unconditional mean and bivariate Ordinary Least Squares (OLS) regressions. Some evidence on the developmental trajectory of cognitive decline in later life suggested that a quadratic age term would be needed to model how these scores change over time.^47^ While we found that including quadratic age terms improved model fit when comparing between- and within-person variance components and AIC and BICs, models that included linear and quadratic or polynomial random effects did not converge on any iteration. Thus, we estimated adjusted models using the most complex model form possible while retaining appropriate random slopes: linear growth curves with individual-level random intercepts and a single linear random slope for individual-level age at a given observation.^48^ All mixed models in this study had unstructured covariance matrices. As in previous growth curve analyses with HRS data,^49^ we centered age at 50 (the sample minimum). Missing data on covariates was managed using Multiple Imputation by Chained Equations. Less than 2.5% of data were missing across all observations. Those who had missing data on any occasion were more often Indigenous, Black, or Hispanic, male, had lower levels of education, lived alone, had less annual income, had more chronic health conditions, more depressive symptoms, and diagnoses of any psychiatric condition. Imputations models adjusted for within-person clustering by including indicators for each participant's ID variable.^50^ Following imputation, we tested the relative efficiency of imputed data, manually inspected the means, frequencies, and distributions of imputed variables, and compared parameter estimates and variance components with unimputed estimates. The relative efficiency of all imputed variables exceeded 99%, no increases in standard error exceeded 20%, and parameter estimates remained consistent relative to unimputed data in fully adjusted models. The results described here are from imputed data, and we report regression coefficients for associations between covariates and initial cognition scores (*b*_*0*_), and rates of change (slopes) in cognition (*b*). This study was ruled exempt from human subjects review by the New York university IRB (IRB-FY2021-5234).

### Role of the funding source

The funder of the study had no role in study design, data analysis, data interpretation, or writing of the report.

## Results

### Sample characteristics

Our inclusion criteria yielded a total sample of 27,327 older adults. IOAs comprised 1.65% of this sample (n = 458), a proportion similar to the total U.S. population.^51^ These data were unbalanced, and the number of observations per participant ranged from 1 to 8. This sample had an average of 4.60 observations across all races, and IOAs had an average of 4.51 observations per participant. Across ethnic categories, we did find differences in the number of time points participants completed. Over three-quarters (77%) of all older adults in this sample contributed at least three observations. However, fewer Indigenous participants completed three or more time points (75%). Exploratory bivariate logistic regression analysis indicated that Indigenous participants had 20.2% lower odds of participating in three or more survey time points than non-Hispanic Whites. However, adjusting this model for educational attainment rendered this relationship nonsignificant. Indigenous participants contributed a total of 2050 outcome time points across the study period.

Evidence of weighted baseline differences across ethnicity is reported in Table 1. Raw descriptive statistics are reported in a Supplementary Table. The average age of the sample at first observation was ∼61 years of age (SD = 9.66), and age ranged from 50 to 107 years old. At first observation, Indigenous, Black, and Hispanic participants were slightly younger than White participants (*p* < 0.001). Across all ethnic groups, women made up just over half of this sample.

**Table 1:** Weighted descriptive statistics at time 1 (N = 27,327).

	Indigenous (n = 458.00)	Black/AA (n = 5411.00)	Hispanic (n = 3713.00)	White (n = 17,745.00)	Total (n = 27,327.00)
*Proportion of total sample*	(1.65%)	(11.43%)	(9.85%)	(77.07%)	(100.00%)
*Age range across study period*	50–97	50–107	50–101	50–102	50–107
*Mean number of timepoints*	4.51	4.33	4.38	4.73	4.60

	Mean (SD) or %	Mean (SD) or %	Mean (SD) or %	Mean (SD) or %	Mean (SD) or %
Age∗∗∗	59.54 (8.05)	59.11 (7.95)	58.87 (7.75)	61.90 (10.04)	61.24 (9.66)
Female∗∗	51.75%	55.30%	50.89%	51.40%	51.80%
Education∗∗∗					
Less than high school	33.99%	22.30%	46.80%	10.95%	16.16%
High school	27.28%	30.09%	22.63%	31.79%	30.62%
Some college	23.28%	28.57%	18.68%	26.30%	25.76%
College+	15.46%	19.04%	11.89%	30.96%	27.46%
Lowest income quartile∗∗∗	26.27%	31.20%	34.24%	12.53%	17.03%
Current Smoker∗∗∗	23.65%	24.52%	15.95%	17.36%	18.14%
Heavy alcohol consumption∗	3.19%	2.41%	3.01%	3.71%	3.49%
Regular physical activity∗∗∗					
Inactive	7.82%	11.27%	8.89%	7.08%	7.75%
Light physical activity	18.10%	20.76%	18.92%	15.09%	16.17%
Moderate physical activity	37.37%	35.56%	36.22%	38.72%	38.09%
Vigorous physical activity	36.71%	32.42%	35.97%	39.10%	37.99%
Lives alone∗∗∗	28.74%	43.60%	26.77%	28.15%	29.79%
Formal volunteering∗∗∗					
None	72.74%	61.46%	77.28%	60.33%	62.31%
<100 h/year	17.20%	22.91%	15.36%	23.66%	22.67%
100+ h/year	10.06%	15.64%	7.36%	16.00%	15.03%
Informal volunteering∗∗∗					
None	41.08%	43.86%	63.77%	34.72%	38.69%
<100 h/year	43.05%	41.99%	27.65%	47.08%	44.55%
100+ h/year	15.87%	14.15%	8.58%	18.20%	16.76%
Unmanaged hearing loss∗∗∗	3.36%	2.27%	2.87%	3.88%	3.59%
High blood pressure∗∗∗	50.34%	65.48%	48.62%	47.65%	49.83%
Diabetes∗∗∗	27.93%	25.39%	27.40%	14.71%	17.40%
Any heart condition∗∗∗	21.08%	17.37%	11.25%	19.17%	18.21%
Any psychiatric diagnosis∗∗∗	22.50%	13.68%	15.01%	18.09%	17.35%
Childhood head injury∗∗∗	11.41%	6.14%	6.07%	12.10%	10.80%
CESD depression score∗∗∗	2.05 (2.35)	1.84 (2.08)	1.97 (2.32)	1.39 (1.95)	1.51 (2.03)
ADRD diagnosis reported∗∗∗^a^	3.38%	0.78%	1.13%	0.85%	0.91%
Total cognition∗∗∗	14.69 (3.94)	14.26 (3.87)	14.27 (3.77)	16.70 (3.75)	16.14 (3.90)

∗*p* < 0.05, ∗∗*p* < 0.01, ∗∗∗*p* < 0.001.

*Note*: Comparisons across race/ethnicity were conducted using one-way ANOVA for continuous variables and Chi-squared tests for categorical variables.

a Respondents who reported ADRD diagnoses at time 1 were included in this sample if they scored above the TICS-m cutpoint for dementias (scores >6 on the 27-point measure).

Indigenous, Black, and Hispanic older adults had significantly lower rates of college completion when compared with Whites. Many Hispanic (46%) and Indigenous (33%) older adults did not graduate from high school. Significantly higher proportions of minoritized older adults fell in the lowest income quartile of this sample compared to Whites. Among Indigenous older adults, just over one-quarter had incomes in the lowest quartile, just over twice the proportion of White older adults (13%).

There were modest differences in health behaviors across ethnicity. About one-quarter of Indigenous and Black participants reported currently smoking, higher than Hispanics and Whites (*p* < 0.001). Only 3.5% of all participants reported engaging in heavy alcohol use, and differences across ethnicity were modest. The overall prevalence of physical inactivity was low across all groups but was slightly more prevalent among Black older adults (11%, *p* < 0.001).

Significantly more Black older adults lived alone than any other group, followed by Indigenous older adults. White older adults reported the highest participation rates in formal volunteering activities; about 40% volunteered with an organization the preceding year. Indigenous and Hispanic older adults reported the lowest rates of formal volunteering activity (*p* < 0.001). However, Indigenous older adults had comparable levels of engagement with informal volunteering activities to Whites and Blacks: about 60% of these respondents engaged in informal volunteering activities the previous year.

There was significant variation across ethnicity in measures of physical and mental health. Between 2% and 4% of all older adults in this sample reported having impaired hearing but did not use hearing aids. Relative to White older adults, Indigenous older adults reported elevated rates of diabetes, heart conditions, depressive symptoms, psychiatric diagnoses, and diagnoses of memory-related conditions. Indigenous and White older adults reported similar rates of head injuries in childhood, about twice the proportion of Black and Hispanic participants. Notably, just over 3% of Indigenous respondents reported having a diagnosis of Alzheimer's disease, dementia, or any memory-related condition at first observation, the highest proportion of any group.

A pattern of inequity across ethnicity was apparent in cognitive functioning at baseline: Indigenous, Black, and Hispanic older adults all scored significantly lower than Whites on total cognition at first observation (*p* < 0.001).

### Regression results

Fully adjusted models indicated that older Indigenous, Black, and Hispanic adults scored significantly lower in total cognition than Whites at age 50 (Table 2). Indigenous older adults scored two points lower in total cognition than White older adults (*b*_*0*_ = −2.01, 95% CI: −2.42, −1.60) and, on average across all observations, declined by 0.82 points in total cognition every 10 years.

**Table 2:** Imputed mixed effect growth curves.

	Adjusted model				Adjusted indigenous-only model^a^			
	Initial level		Rate of change		Initial level		Rate of change	
	b_0_	95% CI	b	95% CI	b_0_	95% CI	b	95% CI
Age in decades (Centered at 50)	16.36∗∗∗	(16.14, 16.57)	−1.85∗∗∗	(−1.95, −1.76)	13.43∗∗∗	(12.29, 14.57)	−1.03∗∗∗	(−1.56, −0.50)
Race (ref. = White)								
Black/AA	−2.78∗∗∗	(−2.92, −2.64)	0.43∗∗∗	(0.35, 0.51)	–	–	–	–
Hispanic	−2.26∗∗∗	(−2.42, −2.10)	0.67∗∗∗	(0.57, 0.76)	–	–	–	–
Indigenous	−2.01∗∗∗	(−2.42, −1.60)	0.34∗∗	(0.11, 0.58)	–	–	–	–
Female (ref. = Male)	0.71∗∗∗	(0.59, 0.83)	0.07∗	(0.01, 0.13)	1.71∗∗∗	(0.81, 2.61)	−0.54∗	(−1.05, −0.03)
Education (ref. ≤ High School)								
High school	1.15∗∗∗	(0.99, 1.31)	0.26∗∗∗	(0.18, 0.34)	1.55∗∗	(0.39, 2.71)	0.09	(−0.52, 0.70)
Some college	2.02∗∗∗	(1.84, 2.20)	0.27∗∗∗	(0.18, 0.35)	2.03∗∗	(0.81, 3.25)	0.50	(−0.21, 1.21)
College or more	3.09∗∗∗	(2.91, 3.27)	0.24∗∗∗	(0.15, 0.33)	2.66∗∗∗	(1.25, 4.07)	0.79∗	(0.01, 1.57)
Lowest income quartile	−0.25∗∗∗	(−0.35, −0.15)	−0.05	(−0.09, −0.01)	0.27	(−0.44, 0.98)	−0.22	(−0.59, 0.15)
Current smoker	−0.72∗∗∗	(−0.84, −0.60)	0.32∗∗∗	(0.24, 0.40)	−0.42	(−0.99, 0.15)	–	–
Heavy alcohol use	−0.40∗∗∗	(−0.64, −0.16)	0.32∗∗∗	(0.18, 0.46)	0.27	(−0.75, 1.29)	–	–
Regular physical activity (ref. = Inactive)								
Light physical activity	−0.03	(−0.17, 0.11)	0.26∗∗∗	(0.20, 0.32)	0.62∗	(0.09, 1.15)	–	–
Moderate physical activity	0.11	(−0.03, 0.25)	0.27∗∗∗	(0.21, 0.33)	0.81∗∗	(0.30, 1.32)	–	–
Vigorous physical activity	−0.00	(−0.14, 0.14)	0.36∗∗∗	(0.30, 0.42)	0.67∗	(0.12, 1.22)	–	–
Lives alone	0.14∗∗	(0.04, 0.24)	−0.17∗∗∗	(−0.21, −0.13)	−0.03	(−0.46, 0.40)	−0.23∗∗∗	(−0.27, −0.19)
Formal volunteering (ref. = None)								
Less than 100 h/year	−0.01	(−0.11, 0.09)	0.15∗∗∗	(0.11, 0.19)	−0.46	(−1.28, 0.36)	0.28	(−0.19, 0.75)
100 or more h/year	0.13∗	(0.01, 0.25)	0.21∗∗∗	(0.15, 0.27)	−0.58	(−1.76, 0.60)	0.75∗	(0.10, 1.40)
Informal Volunteering (ref. = None)								
Less than 100 h/year	−0.00	(−0.08, 0.08)	0.16∗∗∗	(0.12, 0.20)	0.13	(−0.22, 0.48)	–	–
100 or more h/year	−0.07	(−0.19, 0.05)	0.26∗∗∗	(0.20, 0.32)	0.02	(−0.51, 0.55)	–	–
Unmanaged hearing loss	−0.47∗∗∗	(−0.57, −0.37)			−0.43	(−1.19, 0.33)		
High blood pressure	−0.06∗	(−0.12, 0.00)			0.01	(−0.44, 0.46)		
Diabetes	−0.11∗∗∗	(−0.17, −0.05)			0.15	(−0.32, 0.62)		
Any heart condition	−0.10∗∗	(−0.16, −0.04)			−0.17	(−0.70, 0.36)		
Depression symptoms	−0.14∗∗∗	(−0.16, −0.12)	0.02∗∗∗	(0.00, 0.04)	−0.25∗∗∗	(−0.33, −0.17)	–	–
Any psychiatric diagnosis	−0.28∗∗∗	(−0.40, −0.16)	0.02	(−0.04, 0.08)	−0.77∗∗	(−1.32, −0.22)	–	–
Childhood head injury	0.05	(−0.07, 0.17)			0.19	(−0.79, 1.17)		
ADRD diagnosis	−0.78∗∗∗	(−1.09, −0.46)	−0.58∗∗∗	(−0.70, −0.46)	−1.27∗∗	(−2.17, −0.37)	–	–
Random effects								
Age	0.71∗∗∗	(0.65, 0.77)			0.62	(−0.14, 1.38)		
Intercept	2.38∗∗∗	(2.32, 2.44)			2.63∗∗∗	(2.08, 3.18)		
Age and intercept covariance	−0.30∗∗∗	(−0.37, −0.23)			−0.09	(−0.99, 0.81)		
Residual	2.66∗∗∗	(2.65, 2.68)			2.81∗∗∗	(2.71, 2.91)		
Total observations	125,694				2050			

∗*p* < 0.05, ∗∗*p* < 0.01, ∗∗∗*p* < 0.001.

a This model excluded time-varying components for smoking, alcohol use, physical activity, depression symptoms, and psychiatric conditions due to its smaller sample size and increased AIC and BIC values on the inclusion of these time-varying terms.

Adjusting for ethnicity, women had higher age 50 cognition scores (*b*_0_ = 0.71, 95% CI: 0.59, 0.83) and slower rates of decline (*b* = 0.07, 95% CI: 0.01, 0.13) than men. Each additional educational attainment level was associated with higher cognitive scores at age 50 and slower rates of cognitive change over time. Low-income older adults had significantly lower cognition scores at age 50 (*b*_0_ = −0.25, 95% CI: −0.35, −0.15) while controlling for other covariates. However, they did not decline at significantly different rates than those with higher incomes in adjusted models.

Smoking (*b*_0_ = −0.72, 95% CI: −0.84, −0.60) and heavy alcohol use (*b*_0_ = −0.40, 95% CI: −0.64, −0.16) were both associated with lower age 50 cognition scores relative to non-smokers, but positively with rates of change (*b* = 0.32, 95% CI: 0.24, 0.40; *b* = 0.32, 95% CI: 0.18, 0.46). Engaging in regular physical activity of any intensity was positively associated with rates of change in adjusted models: vigorous physical exercise at least once per week had the largest positive association with cognition (*b* = 0.36, 95% CI: 0.30, 0.42).

There were mixed associations in the relationship between social engagement and cognitive functioning. Living alone had a small positive association with cognition scores at age 50 (*b*_0_ = 0.14, 95% CI: 0.04, 0.24). However, it was associated with a faster rate of change (*b* = −0.17, 95% CI: −0.21, −0.13). Formal volunteering was also associated with favorable rates of change: Formal volunteers who engaged in less than 100 h (*b* = 0.15, 95% CI: 0.11, 0.19) and 100 h or more (*b* = 0.21, 95% CI: 0.15, 0.27) had positive cognition slopes relative to those who engaged in no formal volunteering. Formal volunteers who participated in 100 or more hours of volunteering in the last year also had slightly higher cognition scores at age 50 (*b*_0_ = 0.13, 95% CI: 0.01, 0.25) than non-volunteers. Informal volunteering was not associated with higher age 50 cognition scores in any model. However, respondents who engaged in informal volunteering had slower decline rates. Respondents who engaged in less than 100 h (*b* = 0.16, 95% CI: 0.12, 0.20) and 100 h or more (*b* = 0.26, 95% CI: 0.20, 0.32) of informal volunteering activities cognitively declined slower than older adults who engaged in no informal volunteering.

As expected, all chronic physical and mental health conditions were negatively associated with total cognition scores at age 50, and we did not find sufficient variation over time to interact these terms with age. Notably, unmanaged hearing loss was associated with a significant and relatively large difference in cognition scores at age 50 (*b*_0_ = −0.47, 95% CI: −0.57, −0.37).

Finally, our within-group model among only Indigenous respondents (Table 2) revealed that Indigenous women had significantly higher cognition scores at age 50 relative to men (*b*_*0*_ = 1.71, 95% CI: 0.81, 2.61) but that they declined significantly faster as they aged (*b* = −0.54, 95% CI: −1.05, −0.03). Higher levels of educational attainment were associated with significantly higher total cognition scores at age 50, but only having a college degree or more was significantly associated with a slower rate of change in cognition (*b* = 0.79, 95% CI: 0.01, 1.57). Regular physical activity of any intensity was positively associated with age 50 total cognition. Social engagement via living with others and high-intensity formal volunteering was associated with slower rates of change in cognition but not with differences in cognition at age 50. High-intensity formal volunteering was positively associated with rates of change in total cognition (*b* = 0.75, 95% CI: 0.10, 1.40). Among chronic health conditions, only depression, psychiatric diagnoses, and diagnosis of memory-related conditions were negatively associated with cognition at age 50.

## Discussion

To our knowledge, this is the first longitudinal population-based study that explored modifiable factors associated with cognitive health inequities in the HRS data with Indigenous older adults in the United States. Minoritized older adults had significantly lower levels of economic and social resources relative to Whites—such as education, financial assets, and social ties—and lower levels of cognitive health in later life. Indigenous and Black populations had notably fewer resources in these social determinants of health, and their cognitive trajectories began and remained lower relative to Whites and Hispanics.

Historical experiences of structural racism targeted at Indigenous Americans laid the foundation for resource deprivation and health inequities.^10^^,^^52^, ^53^, ^54^, ^55^ Following the colonization of the United States in the 18th century, government policies forced many Indigenous peoples to relocate to low-resource, rural reservations with limited access to education, healthcare, and poor work conditions.^56^ Unfortunately, many Indigenous people experienced medical abuse in varied forms across U.S. history.^57^ These multigenerational traumas can create heightened skepticism of Western medicine and may negatively impact overall and cognitive health outcomes in later life. Given the persistence of structural inequities in modern society, it is unsurprising that Indigenous older adults report high prevalences of chronic health conditions associated with an increased risk of cognitive impairment, like diabetes and hypertension.^58^, ^59^, ^60^, ^61^, ^62^

Cognitive health interventions require multimodal approaches to prevention across the lifespan and cultural attunement to historical and contemporary dynamics of resource hoarding. Long-lasting forms of oppression, such as forced sterilization, Indian residential schools, and broken treaties, have led to a deep-seated distrust of the U.S. government, researchers, and medical institutions in many Indigenous communities.^63^ It is thus critical that we partner with and learn from Indigenous communities when developing interventions, policies, and research agendas designed to improve population health.^64^ This study lends additional credence to equitable access to pathways that preserve cognitive health, such as education in early life and civic engagement in later life.

### Educational equity: a structural origin of cognitive inequities

The protective relationship between educational attainment and cognitive health was large and persistent across the sample.^12^ Our finding that higher levels of educational attainment were associated with slower rates of cognitive decline was unexpected but lends additional credence to the structural origins of inequity posited by the minority stress framework,^65^ cognitive reserve hypothesis,^66^ and the multiplicative benefits of educational equity. Indigenous respondents with college degrees or more scored 2.66 points higher on total cognition at age fifty than those who did not complete high school. Across the 16 years of observation, they experienced a slower decline in cognitive health relative to Indigenous respondents with less education. Nevertheless, only about 15% of all Indigenous participants had college degrees, half of the proportion of Whites. This reflects a missed opportunity for higher education and preserving cognitive health. We suggest it is a critical area for intervention.

Schools remain fraught symbols for many Indigenous communities. How are communities meant to trust “Indian” schools, most of which are run by the federal government or religiously affiliated? It is a well-held belief among Indigenous communities that the original intent of government and religious institutions was to facilitate the extermination of Indigenous cultures and ensure their economic subjugation.^67^ A recent report from ProPublica revealed that the Bureau of Indian Education, the federal agency responsible for more than 180 Indian schools today, delivers poor-quality schooling to students.^68^ As of 2019, only 59% of eligible students graduated from Bureau for Indian Education high schools, 26% lower than the national average from public schools and 15% lower than Indigenous students who attended public schools.^68^ These statistics forecast a public health future that may entrench rather than disrupt enduring cognitive health inequities.

Multigenerational educational traumas likely interact with modern inequities in school quality to create additional barriers to success for Indigenous students and subsequently increase the magnitude of cognitive health disparities in later life. While findings from this study reinforce formal education as a critical protective factor for later-life cognitive health, we consider it equally important that our policy solutions consider the historical, cultural, and individual contexts of Indigenous communities.

### Civic ties: a source of resilience in the context of inequities

Despite evidence of prevalent cognitive health risk factors among this sample of Indigenous older adults, we also found evidence of critical protective factors in later life. Over half of Indigenous respondents regularly volunteered to support their families, loved ones, and communities. Many Indigenous older adults have relatively high levels of engagement with their communities and a strong sense of generativity.^36^^,^^69^^,^^70^ Lower levels of engagement with formal volunteering among Indigenous older adults may be tied to structural and cultural barriers that limit engagement, cultural relevance,^71^ and interest in available programs.^38^ Further exploration is needed on the intensity of volunteering activities in diverse Indigenous communities and the quality and types of opportunities available to them. A recent phenomenological study revealed that Blacks and Latin*e* immigrants characterized civic participation by emphasizing community belonging, religious/spiritual practice, and a way of life.^71^ Future research needs to identify the mechanisms of volunteering with cognition. Volunteering may require data analysis, organizing a community event, or even restoring a community after a natural disaster. Memory, executive processing, focus, decision-making, and emotional regulation are cognitive demands necessary to accomplish these volunteer tasks, yet we do not know the unique impact of social, cognitive, and physical demands on cognition in the context of volunteer activities. Nor are we aware of the variability between volunteer roles and tasks. From a life-course perspective, ensuring resource equity will likely impact cognitive health the most. Nonetheless, this study reinforces the hypothesis that volunteering may gird cognitive health, while controlling for education, income, and other social ties. Federal policies designed to bolster volunteering opportunities within Indigenous communities, such as Title VI of the Older Americans Act, are consistently underfunded, which would limit the number and quality of available volunteer roles.^55^ AmeriCorps Seniors, another federally funded program, recently completed a demonstration project with Native Nations; anecdotal data suggests positive associations with health. Applying rigorous evaluation methods with participants in these national civic programs could reveal the mechanisms of volunteering while considering individual, family, and community resources. This type of research could help direct resources for Indigenous communities to develop, implement, and maintain civic programs to protect cognitive health while preserving Indigenous languages, cultures and histories.

### Limitations

There are several limitations to this exploratory study. First, while we chose to model these cognitive trajectories using a linear form, non-linear models may fit these data better. We elected for a more conservative modeling strategy given that Indigenous older adults were the smallest and youngest racial group in this analysis. Including higher-order random effects with non-linear models prevented models from converging, and we thus elected to proceed with linear models.^48^ Because of this, as estimates approach the upper age limits in this sample, the 95% confidence intervals overlap with the error bands from other groups and reflect the relatively small number of Indigenous peoples who live beyond the age of 85. In future research, this challenge can be managed in two ways: Oversample Indigenous older adults to increase power or employ joint modeling strategies that adjust for the probability of death. The Health and Retirement Study began oversampling Black and Hispanic older adults starting in 2010, but oversampling other racial and ethnic minority groups does not occur. Extending oversampling efforts to older adults who are at high risk of disparate health outcomes in later life, like Indigenous older adults, will be an essential step toward improving equity in gerontological research. Joint modeling strategies may also better model longitudinal estimates for younger, high-mortality populations like this study. Second, this study was observational and thus did not test causal relationships between our variables of interest and cognition. Future research may be able to disentangle causal pathways between these potential mechanisms using experimental or quasi-experimental designs. Third, this study did not account for attrition due to death. This challenge could be addressed in future research by employing joint modeling strategies that adjust for the probability of death. Fourth, while these multivariate models are both parsimonious and complex, including genetic markers associated with later-life cognitive health may better model cognitive trajectories in future research. Finally, causal research is needed to discern the mechanisms that drive the positive association between volunteering and cognitive health. Volunteers tend to have more individual, household, and neighborhood resources than non-volunteers, and these resources may partially explain the associations with cognition.

### Conclusion

This representative longitudinal exploratory study on Indigenous, Black, Hispanic, and White older adults revealed fundamental inequities in structural and individual resources and associations with cognitive health, confirmed previous research^47^^,^^71^ and extended this line of scientific inquiry to Indigenous older adults. Indigenous older adults, like other minoritized populations, are at elevated risk of developing cognitive pathologies in later life. Interventions that shift the focus of preventative interventions away from individual behaviors and toward resolving structural inequities across the lifespan hold promise to eliminate subsequent disparities in later-life cognitive health across race and ethnicity.

## Contributors

CW and EG conceptualized this analysis and collaborated in funding acquisition. CW led data curation and coding and executed formal analysis in collaboration with EG. Only CW had access to raw data (identifiable data) on Indigenous participants, all other data are publicly available at https://hrs.isr.umich.edu/about. EG and CW collaborated on methodology, formal analysis, and validation. CW led project administration and table/figure generation. Both authors contributed to the writing and editing of the manuscript. EG supervised the project and overall investigation by CW. CW had final responsibility for the decision to submit the publication.

## Data sharing statement

These data are publicly available via restricted data access application to the Health and Retirement Study.

## Declaration of interests

CW was supported by stipends from the National Institute on Aging (NIA) and the Grand Challenges of Social Work (GCSW) for research related to this study. CW also received honorariums from the International Indigenous Dementias Research Network and the National Alzheimer's Coordinating Center Data Sovereignty Committee during this study. EG had no competing interests.

## References

[1] Manly J.J., Jones R.N., Langa K.M. (2022). Estimating the prevalence of dementia and mild cognitive impairment in the US: the 2016 health and retirement study harmonized cognitive assessment protocol project. JAMA Neurol.

[2] Nandi A., Counts N., Broker J. (2024). Cost of care for Alzheimer's disease and related dementias in the United States: 2016 to 2060. NPJ Aging.

[3] Administration on Aging (2020).

[4] Mayeda E.R., Glymour M.M., Quesenberry C.P., Whitmer R.A. (2016). Inequalities in dementia incidence between six racial and ethnic groups over 14 years. Alzheimers Dement.

[5] Moon H.E., Kaholokula J.K., MacLehose R.F., Rote S.M. (2023). Prevalence of dementia in American Indians and Alaska natives compared to white, Black, and Hispanic Medicare beneficiaries: findings from the national health and aging trends study. J Racial Ethn Health Disparities.

[6] Clarke A.J., Christensen M., Balabanski A.H. (2024). Prevalence of dementia among Indigenous populations of countries with a very high Human Development Index: a systematic review. Lancet Healthy Longev.

[7] Suchy-Dicey A.M., Domoto-Reilly K., Nelson L. (2024). Epidemiology and prevalence of dementia and Alzheimer's disease in American Indians: data from the strong heart study. Alzheimers Dement.

[8] Apostolou A., Kennedy J.L., Person M.K. (2024). Alzheimer's disease and related dementia diagnoses among American Indian and Alaska Native adults aged ≥45 years, Indian Health Service System, 2016–2020. J Am Geriatr Soc.

[9] Walker J.D., Spiro G., Loewen K., Jacklin K. (2020). Alzheimer's disease and related dementia in indigenous populations: a systematic review of risk factors. J Alzheimers Dis.

[10] Jacklin K., Walker J. (2020). Cultural understandings of dementia in indigenous peoples: a qualitative evidence synthesis. Can J Aging.

[11] de Souza-Talarico J.N., de Carvalho A.P., Brucki S.M., Nitrini R., Ferretti-Rebustini R.E. (2016). Dementia and cognitive impairment prevalence and associated factors in indigenous populations: a systematic review. Alzheimer Dis Assoc Disord.

[12] Livingston G., Huntley J., Liu K.Y. (2024). Dementia prevention, intervention, and care: 2024 report of the lancet standing commission. Lancet.

[13] Payton N.M., Marseglia A., Grande G. (2023). Trajectories of cognitive decline and dementia development: a 12-year longitudinal study. Alzheimers Dement.

[14] Stern Y. (2012). Cognitive reserve in ageing and Alzheimer's disease. Lancet Neurol.

[15] Beam C.R., Kaneshiro C., Jang J.Y., Reynolds C.A., Pedersen N.L., Gatz M. (2018). Differences between women and men in incidence rates of dementia and Alzheimer's disease. J Alzheimers Dis.

[16] Opdebeeck C., Martyr A., Clare L. (2016). Cognitive reserve and cognitive function in healthy older people: a meta-analysis. Neuropsychol Dev Cogn B Aging Neuropsychol Cogn.

[17] Dean E.B., Schilbach F., Schofield H., Barrett C.B., Carter M., Chavas J.-P., Carter M.R. (2018). The economics of poverty traps: poverty and cognitive function.

[18] Guiney H., Keall M., Machado L. (2021). Volunteering in older adulthood is associated with activity engagement and cognitive functioning. Neuropsychol Dev Cogn B Aging Neuropsychol Cogn.

[19] Sabia S., Dugravot A., Dartigues J.F. (2017). Physical activity, cognitive decline, and risk of dementia: 28 year follow-up of Whitehall II cohort study. BMJ.

[20] Deal J.A., Power M.C., Palta P. (2020). Relationship of cigarette smoking and time of quitting with incident dementia and cognitive decline. J Am Geriatr Soc.

[21] Anstey K.J., Mack H.A., Cherbuin N. (2009). Alcohol consumption as a risk factor for dementia and cognitive decline: meta-analysis of prospective studies. Am J Geriatr Psychiatry.

[22] Mayeda E.R., Karter A.J., Huang E.S., Moffet H.H., Haan M.N., Whitmer R.A. (2014). Racial/ethnic differences in dementia risk among older type 2 diabetic patients: the diabetes and aging study. Diabetes Care.

[23] Ou Y.N., Tan C.C., Shen X.N. (2020). Blood pressure and risks of cognitive impairment and dementia: a systematic review and meta-analysis of 209 prospective studies. Hypertension.

[24] Chen L., Lu B. (2020). Cognitive reserve regulates the association between hearing difficulties and incident cognitive impairment evidence from a longitudinal study in China. Int Psychogeriatr.

[25] Wang X.-J., Xu W., Li J.-Q. (2018). Early-life risk factors for dementia and cognitive impairment in later life: a systematic review and meta-analysis. J Alzheimers Dis.

[26] Cherbuin N., Kim S., Anstey K.J. (2015). Dementia risk estimates associated with measures of depression: a systematic review and meta-analysis. BMJ Open.

[27] Zahodne L.B., Sharifian N., Kraal A.Z. (2021). Socioeconomic and psychosocial mechanisms underlying racial/ethnic disparities in cognition among older adults. Neuropsychology.

[28] Zahodne L.B., Manly J.J., Smith J., Seeman T., Lachman M.E. (2017). Socioeconomic, health, and psychosocial mediators of racial disparities in cognition in early, middle, and late adulthood. Psychol Aging.

[29] Proulx C.M., Curl A.L., Ermer A.E. (2018). Longitudinal associations between formal volunteering and cognitive functioning. J Gerontol B Psychol Sci Soc Sci.

[30] Griep Y., Hanson L.M., Vantilborgh T., Janssens L., Jones S.K., Hyde M. (2017). Can volunteering in later life reduce the risk of dementia? A 5-year longitudinal study among volunteering and non-volunteering retired seniors. PLoS One.

[31] Zaninotto P., Batty G.D., Allerhand M., Deary I.J. (2018). Cognitive function trajectories and their determinants in older people: 8 years of follow-up in the English longitudinal study of ageing. J Epidemiol Community Health.

[32] Kail B.L., Carr D.C. (2020). More than selection effects: Volunteering is associated with benefits in cognitive functioning. J Gerontol B Psychol Sci Soc Sci.

[33] Carlson M.C., Erickson K.I., Kramer A.F. (2009). Evidence for neurocognitive plasticity in at-risk older adults: the experience corps program. J Gerontol A Biol Sci Med Sci.

[34] Fried L.P., Carlson M.C., Freedman M. (2004). A social model for health promotion for an aging population: initial evidence on the experience corps model. J Urban Health.

[35] Lewis J.P. (2022). Encyclopedia of gerontology and population aging.

[36] Nelson L.A., Noonan C.J., Goldberg J., Buchwald D.S. (2013). Social engagement and physical and cognitive health among American Indian participants in the health and retirement study. J Cross Cult Gerontol.

[37] Johnson K.J., Lee S.H. (2017). Factors associated with volunteering among racial/ethnic groups: findings from the California health interview survey. Res Aging.

[38] Sundeen R.A., Raskoff S.A., Garcia M.C. (2007). Differences in perceived barriers to volunteering to formal organizations: lack of time versus lack of interest. Nonprofit Manag Leadersh.

[39] Livingston G., Huntley J., Sommerlad A. (2020). Dementia prevention, intervention, and care: 2020 report of the lancet commission. Lancet.

[40] Crimmins E.M., Kim J.K., Langa K.M., Weir D.R. (2011). Assessment of cognition using surveys and neuropsychological assessment: the health and retirement study and the aging, demographics, and memory study. J Gerontol B Psychol Sci Soc Sci.

[41] Ofstedal M.B., Fisher G.G., Herzog A.R. (2005).

[42] Langa K.M. (2020).

[43] Truong Q.C., Cervin M., Choo C.C. (2023). Using network analysis to validate domains of the modified telephone interview for cognitive status. Eur J Clin Invest.

[44] Truong Q.C., Choo C., Numbers K. (2023). Enhancing precision of the telephone interview for cognitive status-modified (TICS-M) using the rasch model. Psychol Assess.

[45] Radloff L.S. (1977). The CES-D scale: a self-report depression scale for research in the general population. Appl Psychol Meas.

[46] Wallace R., Herzog A.R., Ofstedal M.B., Steffick D., Fonda S., Langa K.M. (2000). Study HaR.

[47] Zahodne L.B., Sol K., Kraal Z. (2019). Psychosocial pathways to racial/ethnic inequalities in late-life memory trajectories. J Gerontol B Psychol Sci Soc Sci.

[48] Singer J.D., Willett J.B. (2003).

[49] Lee Y.J., Gonzales E., Andel R. (2021). Multifaceted demands of work and their associations with cognitive functioning: findings from the health and retirement study. J Gerontol B Psychol Sci Soc Sci.

[50] Van Buuren S., Brand J.P., Groothuis-Oudshoorn C.G., Rubin D.B. (2006). Fully conditional specification in multivariate imputation. J Stat Comput Simulat.

[51] U.S. Census Bureau Race and ethnicity in the United States: 2010 census and 2020 census 2021. https://www.census.gov/library/visualizations/interactive/race-and-ethnicity-in-the-united-state-2010-and-2020-census.html.

[52] Hulko W., Camille E., Antifeau E., Arnouse M., Bachynski N., Taylor D. (2010). Views of First Nation elders on memory loss and memory care in later life. J Cross Cult Gerontol.

[53] MacDonald J.P., Barnes D.E., Middleton L.E. (2015). Implications of risk factors for Alzheimer's disease in Canada's indigenous population. Can Geriatr J.

[54] Jacklin K., Pace J.E., Warry W. (2015). Informal dementia caregiving among indigenous communities in Ontario, Canada. Care Manag J.

[55] Whetung C. (2023). Undoing structural racism among indigenous older adults to promote health equity. Generations.

[56] Adelson N. (2005). The embodiment of inequity: health disparities in aboriginal Canada. Can J Public Health.

[57] Jones D.S. (2006). The persistence of American Indian health disparities. Am J Public Health.

[58] Acton K.J., Burrows N.R., Geiss L.S., Thompson T. (2003). Diabetes prevalence among American Indians and Alaska natives and the overall population - United States, 1994-2002 (Reprinted from MMWR, vol 52, pg 702-704, 2003). JAMA.

[59] Hutchinson R.N., Shin S. (2014). Systematic review of health disparities for cardiovascular diseases and associated factors among American Indian and Alaska Native populations. PLoS One.

[60] Browne C.V., Ka’opua L.S., Jervis L.L., Alboroto R., Trockman M.L. (2017). United States indigenous populations and dementia: is there a case for culture-based psychosocial interventions?. Gerontologie.

[61] Cobb N., Espey D., King J. (2014). Health behaviors and risk factors among American Indians and Alaska Natives, 2000-2010. Am J Public Health.

[62] Landen M., Roeber J., Naimi T., Nielsen L., Sewell M. (2014). Alcohol-attributable mortality among American Indians and Alaska Natives in the United States, 1999-2009. Am J Public Health.

[63] Pacheco C.M., Daley S.M., Brown T., Filippi M., Greiner K.A., Daley C.M. (2013). Moving forward: breaking the cycle of mistrust between American Indians and researchers. Am J Public Health.

[64] Blind M., Jacklin K., Pitawanakwat K., Ketcher D., Lambrou N., Warry W. (2023). Training indigenous community researchers for community-based participatory ethnographic dementia research: a second-generation model. Int J Qual Methods.

[65] Forrester S.N., Gallo J.J., Whitfield K.E., Thorpe R.J. (2019). A framework of minority stress: from physiological manifestations to cognitive outcomes. Gerontologist.

[66] Stern Y. (2003). The concept of cognitive reserve: a catalyst for research. J Clin Exp Neuropsychol.

[67] Newland BT (2022).

[68] Woods A. (2020). The Federal government gives native students an inadequate education, and gets away with it. ProPublica. https://www.propublica.org/article/the-federal-government-gives-native-students-an-inadequate-education-and-gets-away-with-it.

[69] Tanaka M.F., Glasser M., Supahan T., Vater J. (2020). Giving back to get back: assessment of native and non-native American perceptions of generativity. J Health Care Poor Underserved.

[70] Lewis J.P. (2021). Aging across cultures: growing old in the non-western world.

[71] Reyes L. (2024). Their words: African American and Latine immigrant older adults (Re)define civic participation. J Gerontol B Psychol Sci Soc Sci.

