# Supplementary material for: Modifiable factors associated with cognitive health trajectories among Indigenous, Hispanic, Black, and White older adults: an exploratory longitudinal panel analysis of the health and retirement study with a focus on Indigenous peoples
Source: Lancet Reg Health Am. 2025 Aug 12;50:101207. doi: 10.1016/j.lana.2025.101207 (PMC12359267; doi:10.1016/j.lana.2025.101207)
Supplement: Supplementary Table [file mmc1.docx]

| Supplementary Table·  *Unweighted Descriptive Statistics at Time 1 (N = 27,327)* | | | | | |
| --- | --- | --- | --- | --- | --- |
|  | Indigenous | Black/AA | Hispanic | White | Total |
|  | n=458  (1·68%) | n=5,411  (19·80%) | n=3,713  (13·59%) | n=17,745  (64·94%) | n=27,327  (100·00%) |
|  | Mean (SD) or n (%) | Mean (SD) or n (%) | Mean (SD) or n (%) | Mean (SD) or n (%) | Mean (SD) or n (%) |
| Age*** | 60·72 (9·17) | 59·29 (8·67) | 58·65 (8·35) | 64·62 (10·97) | 62·68 (10·53) |
| Female*** | 249 (54·37%) | 3279 (60·60%) | 2099 (56·53%) | 9991 (56·30%) | 15618 (57·15%) |
| Education*** |  |  |  |  |  |
| Less than High School | 164 (35·96%) | 1251 (23·22%) | 1752 (47·39%) | 2406 (13·60%) | 5573 (20·47%) |
| High School | 126 (27·63%) | 1671 (31·01%) | 845 (22·86%) | 6020 (34·03%) | 8662 (31·81%) |
| Some College | 105 (23·03%) | 1517 (28·16%) | 686 (18·56%) | 4358 (24·64%) | 6666 (24·48%) |
| College+ | 61 (13·38%) | 949 (17·61%) | 414 (11·20%) | 4904 (27·73%) | 6328 (23·24%) |
| Lowest Income Quartile*** | 137 (29·98%) | 1687 (31·23%) | 1300 (35·14%) | 2503 (14·11%) | 5627 (20·61%) |
| Current Smoker*** | 107 (23·41%) | 1326 (24·51%) | 556 (15%) | 2893 (16·31%) | 4882 (17·87%) |
| Alcohol Consumption*** | 16 (3·51%) | 115 (2·14%) | 102 (2·77%) | 577 (3·26%) | 810 (2·97%) |
| Physical Activity*** |  |  |  |  |  |
| No Physical Activity | 43 (9·39%) | 587 (10·85%) | 322 (8·68%) | 1447 (8·16%) | 2399 (8·78%) |
| Light Physical Activity | 84 (18·34%) | 1116 (20·64%) | 694 (18·72%) | 2916 (16·44%) | 4810 (17·61%) |
| Moderate Physical Activity | 165 (36·03%) | 1956 (36·17%) | 1359 (36·65%) | 7023 (39·59%) | 10503 (38·45%) |
| Vigorous Physical Activity | 166 (36·24%) | 1749 (32·34%) | 1333 (35·95%) | 6355 (35·82%) | 9603 (35·16%) |
| Formal Volunteering |  |  |  |  |  |
| None | 324 (71·68%) | 3281 (61·06%) | 2809 (76·75%) | 10903 (61·72%) | 17317 (63·79%) |
| Less than 100 hours/year | 79 (17·48%) | 1258 (23·41%) | 569 (15·55%) | 3900 (22·08%) | 5806 (21·39%) |
| 100 or more hours/year | 49 (10·84%) | 834 (15·52%) | 282 (7·70%) | 2861 (16·20%) | 4026 (14·83%) |
| Informal Volunteering |  |  |  |  |  |
| None | 205 (45·56%) | 2333 (43·49%) | 2366 (64·70%) | 6951 (39·41%) | 11855 (43·73%) |
| Less than 100 hours/year | 178 (39·56%) | 2262 (42·16%) | 1005 (27·48%) | 7628 (43·25%) | 11073 (40·85%) |
| 100 or more hours/year | 67 (14·89%) | 770 (14·35%) | 286 (7·82%) | 3058 (17·34%) | 4181 (15·42%) |
| Unmanaged Hearing Loss*** | 15 (3·28%) | 120 (2·22%) | 104 (2·80%) | 781 (4·40%) | 1020 (3·73%) |
| High Blood Pressure*** | 240 (52·40%) | 3607 (66·67%) | 1784 (48·07%) | 9060 (51·06%) | 14691 (53·77%) |
| Diabetes*** | 127 (27·73%) | 1412 (26·10%) | 1002 (27%) | 2890 (16·29%) | 5431 (19·88%) |
| Any Heart Condition*** | 98 (21·40%) | 922 (17·04%) | 418 (11·26%) | 4001 (22·55%) | 5439 (19·91%) |
| Any Psychiatric Diagnosis*** | 97 (21·18%) | 740 (13·68%) | 594 (16·01%) | 3175 (17·89%) | 4606 (16·86%) |
| Childhood Head Injury*** | 43 (10·19%) | 309 (6·01%) | 204 (5·79%) | 1838 (11·24%) | 2394 (9·41%) |
| CESD Depression Score*** | 2·01 (2·31) | 1·84 (2·05) | 1·99 (2·34) | 1·39 (1·94) | 1·57 (2·04) |
| ADRD Diagnosis Reported*** | 13 (2·89%) | 46 (0·86%) | 49 (1·33%) | 173 (0·99%) | 281 (1·04%) |
| ** p < 0·05, ** p < 0·01, *** p < 0·001*  Note: Comparisons across race/ethnicity conducted using one-way ANOVA for continuous variables and Chi-squared tests for categorical variables. | | | | | |
